# Impact of acculturation on oral health among immigrants and ethnic minorities: A systematic review

**DOI:** 10.1371/journal.pone.0212891

**Published:** 2019-02-28

**Authors:** Rana Dahlan, Parvaneh Badri, Humam Saltaji, Maryam Amin

**Affiliations:** School of Dentistry, University of Alberta, Edmonton, Alberta, Canada; Centre Hospitalier Regional Universitaire de Tours, FRANCE

## Abstract

**Objective:**

Cultural changes faced by immigrants and ethnic minorities after moving to a host country may have a detrimental or beneficial influence on their oral health and oral health-related behaviors. Therefore, this paper reviews the literature to see the impact of acculturation on immigrants and ethnic minorities’ oral health outcomes.

**Methods:**

We searched seven electronic databases up to January 2018. All cross-sectional and longitudinal quantitative studies that examined associations between acculturation and oral health status and/or oral health behaviors among ethnic minority and immigrant population[s] were included. Study selection, data extraction, and risk of bias assessment were completed in duplicate. The Newcastle-Ottawa checklist was used to appraise the methodological quality of the quantitative studies. A meta-analytic approach was not feasible.

**Results:**

A total of 42 quantitative studies were identified. The studies showed a positive association between acculturation and oral health status/behaviors. The most frequently used acculturation indicators were language spoken by immigrant and ethnic minorities and length of stay at the host country. High-acculturated immigrant and ethnic minority groups demonstrated better oral health outcomes, oral health behaviors, dental care utilization, and dental knowledge.

**Conclusions:**

According to existing evidence, a positive effect of acculturation on oral health status and behaviors was found.

**Practical implications:**

Dental practitioners should be culturally competent to provide the appropriate services and treatments to immigrant and ethnic minorities. Policymakers should also be sensitive to cultural diversities and properly address the unique needs of each group in order to maintain oral health equity.

## Introduction

Global immigration rates have increased dramatically over the past few decades. According to the United Nations, approximately 258 million immigrants in 2017 represented a rise of 49% since 2000 [1]. Upon their arrival in new countries, most immigrants face a number of challenges that negatively influence their quality of life including language and cultural barriers, housing and employment problems, low socioeconomic status, and lack of medical and dental insurance coverage [2–4]. While the prevailing trend for immigrants is to move to a more developed nation than the one they left behind, many arrive in the host countries healthier than their native-born counterparts [3–5]. This difference in health status can be partially explained by the rigorous selection and health screening processes that immigrants are required to undergo [3, 4]. However, after migration, the health of immigrants deteriorates due mainly to changes in lifestyle [6]. The adoption of a westernized diet can be particularly concerning, given its high caloric content that can lead to chronic conditions such as heart disease, diabetes, and hypertension [6].

Immigrant families also exhibit changes in cultural norms when they are exposed to a new culture within the host country. These changes are referred to as “acculturation” [4, 6], which can either be beneficial or detrimental to general and oral health (OH) outcomes [4, 6]. Global indicators such as age at migration, length of stay in the host country, country of origin, and language barrier are used as acculturation proxy measures that could influence immigrants’ acculturation level [4, 7–9]. Interpersonal differences, level of education, age and gender, cultural closeness, can also affect the degree and rate of acculturation [4]. Migration motivation, new country satisfaction, and perceived discrimination, are other important factors in immigrants’ adaptation to the host country socially, culturally, or psychologically [10]. These factors may play a significant role in immigrants’ ability or willingness to either adapt or retain their own culture. Accordingly, acculturation can be divided into four general strategies: assimilation, separation, integration, and marginalization. Assimilation is when individuals prefer to acquire their host country’s cultural identity rather than keep their original one [11–14]. On the other hand, when individuals resist adopting the cultural identity of their new country, preferring instead to preserve their original cultural characteristics, this is referred to as separation [11–14]. The integration strategy, which falls somewhere between assimilation and separation, individuals strive to maintain some of their cultural characteristics in addition to what they acquire from the host cultural identity [11–14]. Marginalization occurs when an individual prefers neither to maintain their original cultural identity nor to interact or acquire any characteristics from the host culture [11–14]. Since acculturation strategies depend on immigrants’ adaptation, Integration is considered as the most preferred strategy for immigrants’ adaptation during their acculturation process, while marginalization is the least one [15, 16]. Furthermore, immigrants’ adaptation is a continuous process that results in different strategies among people and even the same person may go through different strategies in different stages of acculturation [15].

Associations between acculturation and general health [14, 17–19] have shown that highly acculturated immigrants and ethnic minorities had better physical activity, medication adherence, blood pressure level, and mental health compared with low acculturated individuals [18, 19]. Similar correlations have been reported for oral health [6, 19, 20]. For example, among Haitian immigrants in New York City and Vietnamese immigrants in Melbourne, acculturation was inversely related to OH problems [6, 20]. Individuals with high acculturation level showed a low level of decayed teeth and periodontal disease [6, 19]. High acculturation status was also directly proportional to positive behavior adaptability and accessibility to OH care services [6, 20]. Conversely, acculturation may promote some adverse behavioral practices that affect the OH of immigrant and ethnic minorities, such as the adoption of a cariogenic diet [6].

Although a data review of the OH impacts of acculturation has already been published in 2010 [21], new data might have become available that could challenge its conclusions, especially with the growing interest in this field over the past decade. Therefore, the objectives of the present report are to systematically review the impact of acculturation on immigrant and ethnic minority populations OH outcomes and to update previous evidence-based recommendations with new findings.

## Methods

### Protocol and registration

Neither a review registration nor a review protocol was completed. This systematic review is reported in accordance with Cochrane Handbook [22] and the Preferred Reporting Items for Systematic Reviews and Meta-Analyses (PRISMA) statements for reporting systematic reviews of health sciences [23].

### Eligibility criteria

Based on the Participants–Intervention–Comparison–Outcome–Study (PICOS) method [24], we included cross-sectional and longitudinal quantitative studies that 1) examined the association between acculturation and at least one OH status (such as dental caries or periodontal disease) or OH behaviors (such as dental care utilization, brushing, flossing, or diet); 2) included a clearly-defined measure of acculturation either by using proxy measures such as language proficiency, country of origin, age at migration, and length of residence or validated scales like Behavioral and Self-identification Acculturation, The Psychological-Behavioral Acculturation Scale, and Acculturation Rating Scale for Mexican Americans-II; a well-described assessment tool for OH status including DMFT, ICDAS, periodontal attachment loss or self-reported OH status; and self-reported OH behaviors; 3) were conducted with at least one immigrant or ethnic group(s). Excluded were literature reviews, conference abstracts, editorials and, qualitative studies.

### Data sources and search strategy

A comprehensive search was conducted up to January 31, 2018 by using the following electronic bibliographic databases: PubMed (1976–2018), Ovid MEDLINE (1983–2018), ISI Web of Science (1995–2018), Ovid PsychInfo (2008–2018), Sociological Abstracts (1994–2018), Embase (1979–2018), and Cinahl (1989–2018) (S1 Table). The search strategy was developed with the assistance of a specialized health sciences librarian at the University of Alberta, Canada. First, we established the search terms on PubMed; next, we applied and adjusted these search terms on different electronic databases (S1 Table). Manual screening, which is checking all the reference lists of the included studies to find any relevant papers that were missed in the electronic searches, was completed by searching through bibliographies and reference lists of the included papers to determine potential papers that were not found in the electronic search. Finally, a grey literature search was conducted by using Google Scholar and Google search engine.

### Study selection

Two reviewers (RD and PB) independently screened the list of titles and abstracts to identify the potentially relevant papers based on the inclusion criteria. If the abstracts were judged to contain insufficient information, then the full articles were reviewed to decide whether they should be included based on the selection criteria. When a discrepancy in the selection decision occurred, the two reviewers engaged in discussion until a consensus was reached.

### Data extraction and data items

Two reviewers (RD and PB) independently extracted data from the selected papers on the following items: host country, participants’ origins and ages, sampling, sample size, type of study, acculturation measure, association with OH outcomes, and results. Inconsistencies were discussed and resolved between the two authors. Missing or unclear information was sought from the authors of the selected papers. The Newcastle-Ottawa Scale assessed (NOS) the quality of the quantitative studies by scoring three main categories, which are group selection (four items), comparability (one item), and outcome (two items) [25]. A study can be awarded maximum of five stars for selection, a maximum of two stars for group comparability, and a maximum of three stars for outcome categories. The highest methodological quality is indicated by the maximum score; which is 10 points. Studies scored less than 3 are considered low quality, between 3 and 8 are medium quality and above 8 are high quality studies. Although the NOS is easy to apply and an adaptable tool, it has some limitations as there is no manual tool to use as a guide and it’s not validated for cross sectional studies [26, 27].

### Risk of bias in individual studies

Two reviewers (RD and PB) independently assessed the methodological quality of the selected studies by using The Newcastle-Ottawa Scale [25] for cohort, cross-sectional, and case control studies.

### Synthesis of results

Due to the heterogeneity of the included studies, findings were evaluated in a descriptive manner. It was not possible to conduct a meta-analysis.

## Results

### Study selection

**Fig 1:**
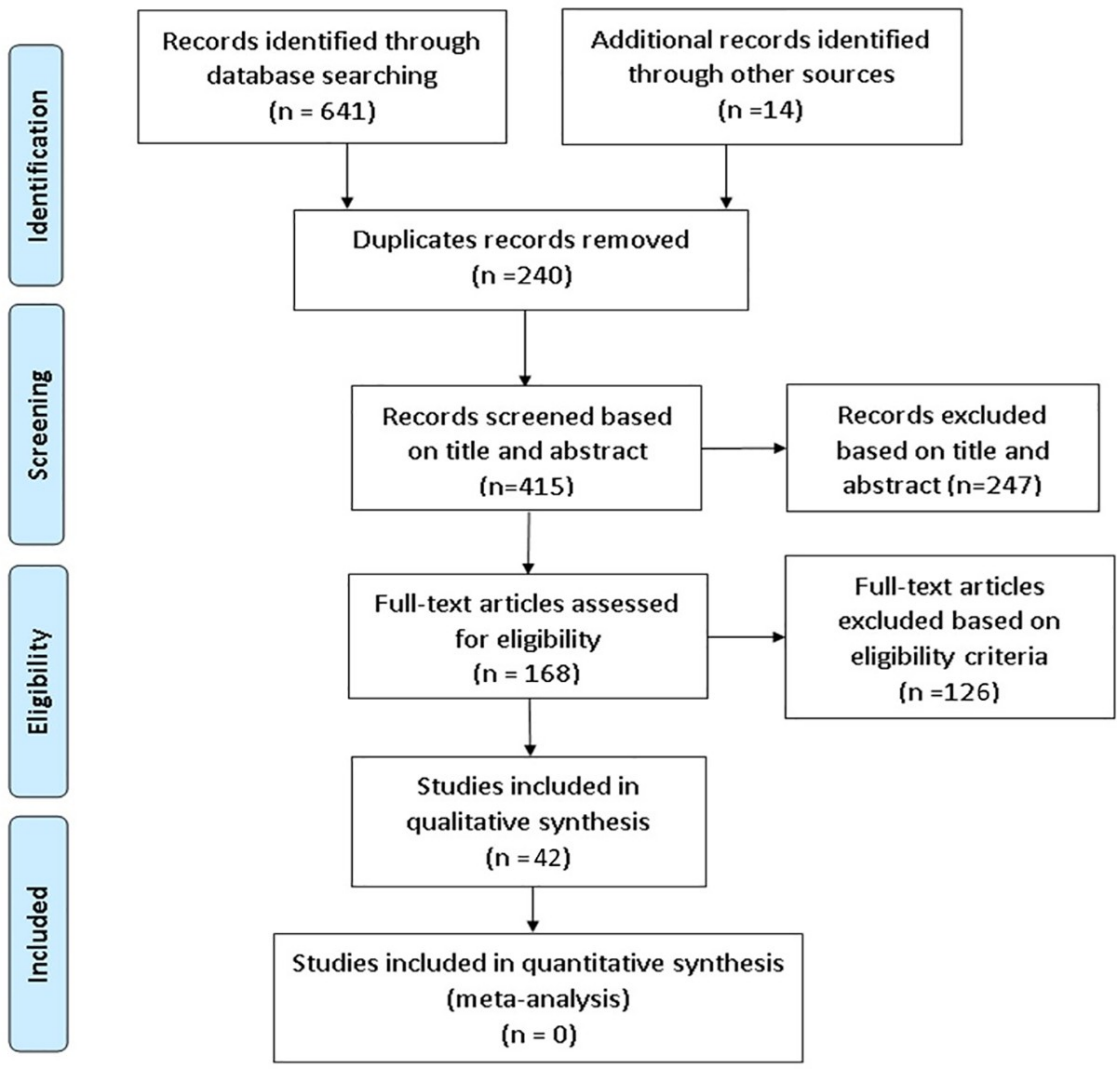
Flow diagram of the literature search according to the PRISMA statement.

The electronic search of seven databases resulted in 641 studies. Of these studies, 168 were found eligible for a full-text review and 30 met our inclusion criteria. With the additional 14 studies found by manual screening, a total of 42 studies were included in our review. The selection process of the included papers is presented in Fig 1.

### Study characteristics

Regarding the study design, 42 were cross-sectional, 1 was cohort, 1 was case-control, and all were written in English. Among the included studies 64% were conducted in the United States and 36% were conducted in other countries including: Canada, Japan, UK, Germany, Norway, China, Australia, New-Zealand, and Sweden. The characteristics of the included studies are presented in Table 1.

**Table 1:** Characteristics of included studies.

Author Year	Host Country & Participants	Age	Study Type and Sample	Acculturation Measure	Association with Oral Health Outcome	Results
Cruz et al.[6] 2004	USA—425 Haitian immigrants	Over 18 years	Cross sectional Convenience	Behavioral and self-identification acculturation scale	NIDCR* diagnostic criteria	• High acculturation: [–] tooth decay; [–] missing teeth; [–] periodontal disease; [0] dental caries experience [+] access to preventive or restorative services better oral heath behaviour adaptability • Length of stay: [+] tooth decay; [+] missing teeth; [0] periodontal health
Finlayson et al.[57] 2010	USA—213 Haitian immigrant families	adults:18–55 years children: >18 years	Cross sectional Random	The Acculturation Rating Scale for Mexican- Americans; Language proficiency	Dental care utilization	• Language proficiency: dental care utilization
Gao et.al.[29] 2014	China– 122 Indonesian domestic helpers	20–59 years	Cross sectional- Cluster Random	Proficiency in local languages	Oral health behaviors; Knowledge of dental caries etiology; Dental caries; Perio-disease	• Local Language proficiency and High acculturation: [+] oral health behaviors; [+] knowledge of dental caries etiology dental caries; [0] periodontal disease; [0] oral health diseases
Geltman et al.[30] 2013	USA—439 Somali adults living in Massachusetts	18 years or older	Cross sectional—purposive	Revised Haitian Acculturation Scale	Tooth decay; Periodontal disease; Dental care utilization; Oral health behavior	• High acculturation: [–] tooth decay; [–] periodontal disease; [+] dental care utilization [0] Oral health behaviors
Geltman et al.[58] 2014	USA—439 Somali adults living in Massachusetts	18 years or older	Cross sectional Convenience +.052.	Revised Haitian Acculturation Scale	Tooth decay; Periodontal disease; Dental care utilization; Use of preventive dental care	• High acculturation level: [+] use of preventive dental care; [–] tooth decay [–] periodontal disease; [+] dental care utilization • Language proficiency: [+] preventive visits
Riley et al.[43] 2008	USA—911 Hispanic immigrants	18 years or older	Cross sectional—Random	Language nativity; cultural identification	Orofacial pain; Regular dentist visits	• Use of English language: [+] healthcare visit for orofacial pain; [+] having a regular dentist; [–] orofacial pain, difficulty eating, sleeping, depression • Nativity: [–] orofacial pain, sleep difficulty • Hispanic culture identification: [–] having a regular dentist visit
Mariño et al.[20] 2001	Australia– 147 Vietnamese immigrants	18 years or older	Cross sectional-Convenience	The Psychological-Behavioral Acculturation Scale	Tooth decay; Dental care utilization; Oral health knowledge; Oral health behavior	• High acculturation: [–] tooth decay; [+] dental care utilization; [+] knowledge of ways of preventing dental caries oral health behaviour • Psychological acculturation: [+] dental visit • Medium level of psychological acculturation: [+] DMFS scores; [–] oral health knowledge • Behavioral acculturation: [–] DMFS
Maupome et al.[59] 2016	USA—301 Latino immigrants	18–70	Cross sectional-Convenience	Psychological-Behavioral Acculturation Scale	Number of months since last dental visit; Main reason for last dental visit	• Behavioral acculturation: [+] recent dental care utilization • Psychological acculturation: [+] planned and preventive dental care
Mejia et al.[44] 2011	USA—10450 Hispanic, non-Hispanic, Asian	6–8 years	Cross sectional–Random cluster	Language spoken at home and school; percent of English language learners	Lack of sealants	• Language spoken at home other than English: [–] dental sealant • Percent of English language learners: [–] dental sealant
Ogami et al.[60] 2016	Canada– 48 Japanese	18 years or older	Cross sectional—Convenience	East Asian Acculturation Measure	Hiroshima University Dental Behavioral Inventory for oral health behaviors/attitudes	• Marginalization: [–] oral health behaviors and attitudes • Separation: [+] oral health behaviors and attitudes
Otsuru et al.[61] 2006	Japan- 244 Asian and Latino	18 years or older	Cross sectional—Convenience	The Psychological Behavioral Acculturation Scale	Dental caries; Periodontal disease; Dental care utilization	• Low acculturation: [–] oral health status; [+] dental caries; [+] periodontal disease; [–] dental care utilization; [+] brushing frequency
Schluter et al.[62] 2017	New Zealand- 1,477 mothers 1,376 children Pacific Island	6.8–15.4 years	Cohort- Convenience	Berry’s bi-directional model framework; general ethnicity questionnaire	Treatment need; Dental caries; Periodontal disease	• Assimilation: [–] treatment need • Separators: [+] treatment need • Higher Pacific orientation: [+] treatment need • Acculturation: [0] Dental caries; [0] Periodontal disease • Assimilators and integrators: [+] Oral health behaviors
Solis et al.[45] 1990	USA– 5411 Hispanics	20–74 years	Cross sectional—Random	Language proficiency; Ethnic identification	Recency of dental visit	• Use of English language: [+] Recency of dental visit • Ethnic identification: [0] Recency of dental visit
Spolsky et al.[31] 2000	USA– 240 Hispanics	over 18 years	Cross sectional—Convenience	Language proficiency	Dental caries; Periodontal disease	• Language proficiency: [+] Oral Health Status Index
Su et al.[46] 2012	USA—966 Hispanic	18 years or older	Cross sectional—random	Language proficiency; Length of stay	Dental care utilization	• High acculturation: [+] dental care utilization in Mexico by US residents • Language proficiency: [–] cross the border for Mexican health services • Length of stay: [–] cross the border for Mexican health services • First generation vs later generations of Mexican immigrants: [+] cross the border for Mexican health services
Jaramillo et al.[28] 2009	USA—21,958 Hispanic immigrants	18 years or older	Cross sectional-Random	Language proficiency	Dental visit within the past 12 months	• Language proficiency: [+] Dental visit in the past 12 months
Ebin et al.[47] 2001	USA—609 Latino adolescents	11–19 years	Cross sectional- Convenience	Country of birth; Language proficiency	Frequency of brushing; Dental visit within the past 12 months	• Country of birth: Dental visit within the past 12 months [+] Frequency of brushing. • Language proficiency: [+] Dental visit within the past 12 months; [+] Frequency of brushing
Ismail et al.[63] 1990	USA—2289 Latino adolescents	12–74 years	Cross sectional -stratified probability	Acculturation index [modified Cuellar scale; Language Ethnic identification	Dental caries; Periodontal disease; Dental care utilization	• High acculturation: [–] dental caries; [–] missing teeth; [–] filed teeth [–] gingivitis and periodontal pocketing; [+] dental care utilization; [+] preventive dental care
Cruz et al.[4] 2009	USA—1318 Chinese, Dominicans, Haitians, Asians, Indians, Puerto Ricans, Hispanics	18–65 years	Cross sectional-Purposive	Country of origin; Age at immigration; Length of Stay; Language preference	Caries; Periodontal disease; Dental care utilization; Oral health behavior	• Length of stay: [–] dental caries; [0] periodontal disease; [+] dental care utilization; [0] oral health behavior • Age at immigration: older age at immigration; [+] dental caries; [+] periodontal disease; [+] treatment need; [0] oral health behavior • Language proficiency: [0] dental caries; [0] periodontal disease; [0] dental care utilization; [0] oral health behavior
Lee et al.[48] 2017	USA—2289 Chinese, Korean, Vietnamese	18 years or older	Cross sectional-Convenience	The Suinn-Lew Asian Self Identity acculturation scale; length of stay	Dental care utilization	• Acculturation: [+] dental care utilization • Length of stay: [+] dental care utilization
Davis et al.[64] 2017	USA—277 Mexican immigrants	20–60 years	Cross sectional-Purposive	12-item ARSMA-II** acculturation scale	Participants’ expectations and perceptions of a dental visit; Dental outcomes	• Low acculturation: [–] service expectation; [0] service perception; [–] dental outcomes
Luo et al.[49] 2017	USA—1,458 Hispanic,Non-Hispanic	30 years or older	Cross sectional-Random	Language proficiency; length of stay	Self-rated oral health and clinically diagnosed periodontitis	• Language proficiency: [–] periodontitis; [+] self-rated oral health • Length of stay: [–] periodontitis; [0] self-rated oral health
Graham et al.[32] 2005	USA– 810 Hispanic	18 years or older	Cross sectional-Random	Primary language spoken at home	Regular dentist visit “dental home”	• Use of English language: [+] Having a dental home
Akresh et al.[42] 2009	USA- 6135 Hispanic/Asian	average is 40 years	Cross sectional- Random	Length of Residence; Language proficiency	Dental care utilization	• Length of residence and having dental insurance coverage: [+] dental visit • English proficiency and income in Hispanics: [+] dental visit • English proficiency in Asians: dental visit
Bissar et al.[33] 2007	Germany– 570 Poland, Turkey	12–14	Cross sectional- Random	Country of birth	Dental caries	• Born in German: [–] DMFT
Quandt et al.[50] 2007	USA—79 children, 108 mothers,102 fathers Hispanic	Children: 13 years Mothers: 27.7 years	Cross sectional-Convenience	Language proficiency; Country of birth; Length of residence	Dental visit in the last year; Oral health rated by mother; Use of dental services	• Local-born children: [+] dental visit in the last year; [+] oral health rated by mother • Mothers’ language preference & length of residence: [0] use of dental services of any family member; [0] self-rated oral health
Selikowitz et al.[34] 1986	Norway– 160 Pakistani immigrants	20 years or older	Cross sectional- Convenience	Length of stay	Dental service utilization; Beliefs about the consequences of dental disease; Knowledge about dental disease etiology	• Length of stay: [0] dental service utilization; [0] Knowledge about dental disease etiology • Length of stay and dental behavior: [+] among women indicating high acculturation level than men • Belief about consequences of dental disease: [+] dental service utilization • Knowledge about dental disease etiology: [0] dental service utilization
Swoboda et al.[35] 2006	USA—733 Asians, Hispanics	60–75 years	Cross sectional—Convenience	Length of stay	Oral health-related quality of life	• Length of stay: [+] oral health-related quality of life
Ugur et al.[51] 2002	Germany– 532 Turkish immigrants	Older than 12 years	Cross sectional- Convenience	Language proficiency; Length of stay	Dental service utilization	• Language proficiency: [+] Dental service utilization • Length of stay: [+] Dental service utilization
Yu et al.[52] 2001	USA—5644 Asians, non-Hispanics, Hispanics	11–21 years	Cross sectional—Random	Language spoken at home; Country of birth	Dental visits	• Language spoken at home: [–] dental visit • Local-born children and parents: [+] dental visit
Watson et al.[65] 1999	USA—142 Hispanic immigrants	2–5 years	Cross sectional- Convenience	The acculturation scale [measures changes in language use]	Dental caries	• Mother’s length of residence in the USA: [–] dental caries in children • Mother’s use of English language: [0] dental caries in children
Wu et al.[36] 2005	USA—477 Chinese and Russian immigrants	60 years and older	Cross Sectional—Convenience	Length of stay	Dental visits	• Length of stay: [0] dental visits among Russian elders; [+] dental visits among Chinese immigrant’s elders
Werneck et al.[53] 2008	Canada– 104 Portuguese-speaking immigrants	Children 4 years or younger	Case Control- Convenience	Parent’s country of origin; Parents’ age at immigration	Early childhood caries	• Parent’s country of origin [Children of mothers from non-European countries, Brazil and Angola]: [+] early childhood caries • Parents’ age at immigration [older age at immigration]: [+] early childhood caries.
Mikami et al.[37] 1999	UK—162 Japanese immigrants	3–12 years	Cross sectional- Random	Country of birth	Use of dental services; Parental knowledge about cause/prevention of dental caries	• Born in United Kingdom: [+] dental visit, [+] parental knowledge concerning the cause and prevention of dental caries, [0] oral health behaviors
Stewart et al.[67] 2002	USA—6324 Hispanic immigrants	Older than 17 years	Cross sectional- Random	Mexican-American acculturation index	Dental care	• High Acculturation:[+] dental care in the past 5 years in all Hispanic groups [+] dental care in the past 2 years in Cuban-American and Puerto Ricans
Locker et al.[38] 1998	Canada– 721 Europeans, Africans, Asians	13–14 years	Cross sectional—Random	Length of stay	Oral health status; Annual dental visit	• Length of stay: [+] oral health status, [–] caries, [–] calclus, [–] gingivitis [–] treatment need, [+] annual dental visit
Selikowiz et al.[39] 1987	Norway– 160 Pakistani immigrants	20 years and older	Cross sectional—Convenience	Length of stay	Subgingival calculus; pocket depth	• Length of residence: [–] subgingival calculus, [–] pocket depth
Lai et al.[54] 2007	Canada– 1,537 Chinese immigrants	65 years or older	Cross sectional—Random	Length of stay; Country of origin; Language proficiency	Dental care utilization	• Length of stay: [+] dental care utilization • Country of origin: [+] dental care utilization • Language proficiency: [0] Dental care utilization
Nurko et al.[55] 1998	USA– 130 Hispanic immigrants	3–16 years	Cross sectional- Convenience	Country of birth; Language use	Dental visits; Dental caries	• Born in United States: [+] dental visit; [–] dental caries • Use of English Language: [+] dental visit; [–] dental caries
Bedi et al.[40] 1989	UK– 643 Asian immigrants	5 years	Cross sectional—Convenience	Mother’s language proficiency	Children’s’ dental caries; Children’s’ oral hygiene	• Mother’s Language Proficiency: [–] children’s dental caries, [+] children’s oral hygiene
Jacobsson et al.[41] 2005	Sweden- 143 immigrant adolescents	15 years	Cross sectional- Convenience	Age at immigration	Dental caries	• Age at immigration; the younger the children were at immigration:[–] dental caries
Silveira et al.[56] 2018	USA—13,172 Hispanic immigrants	18–74 years	Cross sectional- Random	The Acculturation Scale for Hispanics; generation; Birthplace; Years of residence	Oral health related quality of life	• Higher generation: [0] food restriction compared with those who were first generation • U.S. birthplace, length of residence U.S., and high language acculturation: [0] food restriction • High social acculturation: [–] doing usual Jobs/attending school

- NIDCR*: National Institute of Dental and Craniofacial Research diagnostic criteria

- ARSMA **: The Acculturation Rating Scale for Mexican Americans

- DMFS: Decayed- Missing-Filled surfaces

- DMFT: Decayed- Missing-Filled Teeth

- [+] positive correlation; [–] negative correlation; [0] no correlation

The sample size of the included studies ranged from 12 to 21,958 participants [28]. Acculturation level was assessed through the following different measures: one proxy measure only such as language proficiency, country of origin, age at migration, and length of residence[28–42]; multiple proxy measures[4, 42–56]; or certain scales.[6, 20, 33, 48, 56–65]

### Acculturation and oral health

#### Dental caries

Eighteen studies determined the effect of acculturation or its proxies on dental caries [4, 6, 20, 29–31, 33, 38, 40, 41, 50, 53, 55, 61–65]. Among these studies, 5 reported that high acculturated immigrants and ethnic minorities had decreased number of teeth with dental caries [6, 20, 30, 62, 63] and 2 studies showed that low acculturation resulted in high rate of tooth decay [61, 64]. Age at migration was also reported to affect dental caries status [4, 41, 53]: the older the migrants, the worse their dental caries status [4, 53]. In addition, younger immigrants’ children and those who were born in the host country reported a lower level of dental caries than their counterparts [33, 41, 50, 55]. While host language proficiency was inversely associated with dental caries level in 3 studies [31, 40, 55], it was not significantly correlated with caries level in 2 other studies [29, 65].

#### Periodontal disease

The association between acculturation and its attributes and periodontal disease was assessed by 13 studies [4, 6, 29–31, 38, 39, 49, 58, 61–64]. Among these studies, 5 reported a positive association [6, 58, 61, 63, 64]. and 1 reported no significant association between acculturation and periodontal disease [62]. Length of time living in the host country was positively associated with the rate of periodontal disease in 2 studies [38, 49], but it was not significantly associated in 2 other studies [4, 6]. Similarly, host language proficiency was found to be positively associated with periodontal disease in 2 studies [31, 49] but not significantly associated in others [4, 29]. Periodontal disease rate was higher among immigrants and ethnic minorities who were older than 44 years at the time of imigration in 1 study[4], but it was not significantly associated with the age at migration in another study [6]. Country of origin was also correlated with periodontal disease in 1 study [4].

#### Orofacial pain

The association between acculturation attributes and orofacial pain was examined in 1 study [43]. High acculturated Hispanic immigrants more frequent usage of health and dental care for orofacial pain and symptoms [43]. Interestingly, nativity or longer time of residency and English language proficiency were negatively associated with orofacial pain, eating problems, sleeping difficulty, and depression, while recent immigrants had fewer sleep problems [43].

#### Oral health knowledge and behaviors

Five papers reported that immigrants and ethnic minorities with high acculturation level, local language proficiency, and long period of residency had better knowledge of the etiology of caries and periodontal disease, as well as better understanding of how to prevent dental caries [20, 29, 34, 37]. In addition, among 12 studies investigating the association between acculturation and OH behaviors [6, 20, 29, 37, 40, 44, 47, 58, 60, 62], 2 showed significant associations between high acculturation and healthier behaviors including frequent brushing[6] and mouth rinsing, while 2 studies reported no significant association between these variables [20, 58]. One study reported that separators exhibited better OH behaviors than marginalized individuals [11–13, 60]. On the contrary, more brushing frequency was seen among assimilators and integrators [11–14, 62]. Living in a new country was associated with better OH behaviors and adaptability [29]; however, place of birth was not significantly correlated with any oral health-related behaviors [37]. Mother’s language proficiency was also associated with enhanced oral hygiene practices [40] and use of sealant [44] for immigrants’ children, but another study [47] found no association between host country’s language use and OH-related behaviors. Length of residency was reported by some immigrants to be associated with increased consumption of sugary foods and drinks.

#### Dental services utilization

The relationship between acculturation characteristics and dental care utilization was examined in 27 studies [4, 6, 20, 28, 30, 34, 36–38, 42, 45–48, 50–52, 54, 55, 57–59, 61, 63, 66, 67]. Language proficiency and length of living in the host country were the most effective factors influencing dental visits [28, 30, 34, 36, 38, 42, 45, 48, 50–52, 54, 55, 57, 63, 66]. Six studies reported that local language proficiency was positively associated with better dental care utilization [30, 42, 43, 45, 51, 55], and 1 study reported that the continuous use of first language in the host country was negatively related to routine dental visits [52]. The absence of an association between language proficiency and dental visits was found in 2 studies [28, 50]. Seven studies reported that length of stay in the host country was associated with better dental care utilization [36, 38, 42, 48, 51, 54], whereas 2 studies showed no significant association [34, 50]. High acculturated individuals visited dentists regularly and more often than their low acculturated peers [4, 6, 20, 30, 46, 48, 58, 61, 67]. While psychological acculturation was associated with planned and preventive dental care, behavioral acculturation was correlated with recent dental care utilization [59]. Immigrant and ethnic minority groups who showed less identification with their own culture [43] and immigrants’ children who were born in the host countries revealed more dental care utilization [29, 42, 50, 55]. The influence of acculturation upon immigrants’ dental service expectations and perceptions was also examined [64]. Low acculturated individuals exhibited a lower level of service expectations than their high acculturated counterparts, and the perception of service did not differ between the acculturation levels [64].

#### Oral health-related quality of life

The impact of acculturation on oral health-related quality of life was explored by 2 studies. Both studies reported that oral health-related quality of life was significantly associated with the length of living in the host country and high social acculturation [35, 56].

### Risk of bias in the included studies

Overall, the studies included in this systematic review attained medium–high methodological quality, according to the grading method used [25]. Table 2 presents the quality assessment of included paper.

**Table 2:** Critical appraisal for quantitative studies.

Author Year	Selection (Max 5 stars)				Comparability (Max 2 stars)	Outcome (Max 3 stars)	
	1. Representativeness of the sample	2. Sample size	3. Non–respondents	4. Acculturation tool	1.Participants in outcome groups are comparable	1. Assessment of the outcome	2.Statistical test
	a) Truly representative of the average in the target population.(all participants or random sampling)* b) Somewhat representative of the average in the target population.(non random sampling)* c) No description of the sampling strategy.	a) Justified and satisfactory. * b) Not justified.	a) Comparability between respondents and non-respondents characteristics is established, and the response rate is satisfactory. * b) The response rate is unsatisfactory, or the comparability between respondents and non-respondents is unsatisfactory. c) No description of the response rate or the characteristics of the responders and the non-responders.	a) Validated measurement tool. ** b) Non-validated measurement tool, but the tool is available or described.* c) No description of the measurement tool.	a) The study controls for the most important factor (select one). * b) The study control[s] for any additional factor. **	a) Independent masked. ** b) Self report. * c) No description.	a) Clearly described and appropriate, and the measurement of the association is presented, including confidence intervals and the probability level (p value). * b) The statistical test is not appropriate, not described or incomplete.
Cruz et al.[6] 2004	b*	*	c	**	**	**	*
Finlayson et al.[57] 2010	a*	*	c	**	**	**	*
Gao et al.[29] 2014	a*	*	c	*	**	**	*
Geltman et al.[30] 2013	b*	*	c	**	**	**	*
Geltman et al.[58] 2014	b*	b	c	**	**	**	*
Riley et al.[43] 2008	a*	*	c	*	**	*	*
Mariño et al.[20] 2001	b*	b	c	**	**	**	*
Maupome et al.[59] 2016	b*	b	c	**	**	*	*
Mejia et al.[44] 2011	a*	b	c	*	**	**	*
Ogami et al.[60] 2016	b*	*	c	**	**	*	*
Otsuru et al.[61] 2006	b*	*	c	**	**	**	*
Schluter et al.[62] 2017	b*	*	c	**	**	**	*
Solis et al.[45] 1990	a*	*	c	*	**	*	*
Spolsky et al.[31] 2000	b*	b	c	*	**	**	*
Su et al.[46] 2012	a*	*	c	*	**	**	*
Jaramillo et al.[28] 2009	a*	b	c	*	**	*	*
Ebin et al.[47] 2001	b*	b	c	*	**	*	*
Ismail et al.[63] 1999	a*	*	c	**	**	**	*
Cruz et al.[4] 2009	b*	b	c	*	**	**	*
Lee et al.[48] 2017	b*	b	c	**	**	*	*
Davis et al.[64] 2017	b*	b	c	**	*	*	*
Luo et al.[49] 2017	a*	*	c	*	**	**	*
Graham et al.[32] 2005	a*	b	c	*	**	*	*
Akresh et al.[42] 2009	a*	b	b	*	*	**	*
Bissar et al.[33] 2007	b*	b	c	c	*	**	*
Quandt et al.[50] 2007	b*	b	c	**	**	**	*
Selikowitz et al.[34] 1986	b*	b	c	c	**	**	*
Swoboda et al.[35] 2006	b*	*	c	*	**	**	*
Ugur et al.[51] 2002	a*	*	c	*	**	**	*
Yu et al.[52] 2001	b*	b	c	c	**	**	*
Watson et al.[65] 1999	b*	b	c	*	**	*	*
Wu et al.[36] 2005	b*	b	a*	*	**	**	*
Werneck et al.[53] 2008	a*	b	c	c	*	**	*
Mikami et al.[37] 1999	a*	b	c	c	**	*	*
Stewart et al.[67] 2002	a*	b	c	c	**	**	*
Locker et al.[38] 1998	b*	b	c	c	**	**	*
Selikowitz et al.[39] 1987	a*	b	c	c	**	**	*
Lai et al.[54] 2007	b*	b	c	c	**	**	*
Nurko et al.[55] 1998	b*	b	c	c	**	**	*
Bedi et al.[40] 1989	b*	b	c	c	**	**	*
Jacobsson et al.[41] 2005	a*	b	c	c	**	*	*
Silveira et al.[56] 2018	b*	*	c	**	**	**	*

A study can be awarded one star “*” or a maximum of two stars “**” (representing “yes”) for each numbered item within the selection, comparability, and outcome categories.

## Discussion

The association between acculturation and health, in general, and oral health (OH), in particular, has received increased attention in the past decade because of growing migration worldwide. Therefore, this paper is considered as an extention of the previous systematic review as we systematically reviewed the existing reports on the impact of acculturation and its attributes on OH outcomes of immigrants and ethnic minorities [21]. overall, acculturation has been proven to positively influence dental services utilization and OH behaviors of migrants such as brushing frequency and increased flossing. Acculturation was also associated with immigrant and ethnic minoritys’ improved OH status, improved OH knowledge, and reduced orofacial pain.

The panel for updating guidance for systematic reviews (PUGs) consists of “review authors, editors, statisticians, information specialists, related methodologists, and guideline developers met to develop guidance for people considering updating systematic reviews” [68]. According to the (PUGs) an update of a systematic review is defined as: “…a new edition of a published systematic review with changes that can include new data, new methods, or new analyses to the previous edition” [68]. A similar systematic review was conducted by Gao and McGrath in 2010 [21]. Although the evidence reported by the this review was relatively comprehensive at that time, much more knowledge has been added to the literature since then. The increase in the number of papers in this review proves a greater attention of researchers to this topic including the application of acculturation scales that were not used by the older studies included in the previous review [21] like the East Asian Acculturation Measure [60], Berry’s bi-directional model framework; general ethnicity questionnaire [62], Suinn-Lew Asian Self Identity acculturation scale [48], Acculturation Rating Scale for Mexican Americans (ARSMA)-II [64], and the Acculturation Scale for Hispanics [56]. Furthermore, in the present review, the New Castle Ottawa (NOS) checklist was used for quality assessment of nonrandomized studies. This tool has a valid content and inter-rater reliability [25]. The quality assessment tool used in the previous review was non-validated and developed based on guidelines proposed by previous authors [21]. In addition, long time has elapsed since the previous review search ended in January 2010.

In this paper, we have systematically reviewed the existing reports on the impact of acculturation and its attributes on OH outcomes of immigrant and ethnic minority populations and found that, overall, acculturation has been proven to positively influence dental services utilization and OH behaviors such as brushing frequency and increased flossing. Acculturation was also associated with immigrants’ improved OH status, improved OH knowledge, and reduced orofacial pain.

Thirty two of the included studies conducted a multivariate analysis to explore the association between different variables with adjusting some socioeconomic and demographic factors. While interesting, none of the included studies examined the relationships between the acculturation indicators themselves like the possible relationship between time since immigration and language proficiency; however, age was reported to have the most significant effect on dental care utilization in one study [69]. The potential for collinearity between age and age at immigration and between age and length of stay in the United States was also examined in another study and no correlation was found between years in the United States and age at immigration [4].

The papers reviewed in this study used different measures of acculturation. Acculturation proxies such as length of living in the host country, age at migration, language proficiency, or country of origin were used by 25 studies (60%), certain scales were solely used in 12 studies (28%), and certain scales combined with proxy measures were used in 5 studies (12%). The main scales used included the Psychological-Behavioral Acculturation (P-BAS), [61, 69]Acculturation Rating Scale for Mexican Americans (ARSMA) [57], or (ARSMA-II) [64], which consisted some questions about language use and preference, ethnic and cultural identity, ethnic interaction, and values. However, the overreliance on acculturation proxies used by the majority of the studies has caused inconsistencies among their findings mainly because these proxies are unidimensional in nature and therefore reveal only one direction of findings [70, 71]. In other words, unidimensional proxies are unable to explain the extent to which immigrants retain their own culture or adapt to their host culture [71–73]. Furthermore, proxy measures give only a snapshot of immigrants’ cultural changes rather than presenting acculturation as a process [70–72], and they do not consider the psychological domain of acculturation [71, 74]. Therefore, the use of proxies instead of validated measures across most of the reviewed papers led to methodological heterogeneity that precluded us from conducting a meta-analysis.

Host language proficiency, one of the acculturation proxies used by 19 reviewed papers, was found to be significantly associated with improved OH knowledge [20, 29], oral hygiene practices [40], dental attendance [30, 42, 45, 51, 52, 55], preventive services utilization [30], and OH outcomes such as dental caries and periodontal disease [31, 49, 55]. Similar to our findings, limited English language proficiency was associated with lower use of necessary mental health care services and general health care utilization [75, 76]. These findings reveal that language proficiency is one of the most influential behavioral acculturation indicators. It is possible that individuals who speak the host country’s local language gain more confidence which allows them to socialize with native people. In turn, this may lead to immigrants’ increased awareness of OH knowledge and available services. Therefore, providing culturally appropriate services are crucial for culturally and linguistically diverse immigrants to overcome certain barriers [77]. For example, cultural competency training for health-care providers and presence of an interpreter has significantly increased health and dental care utilization, improved patients’ outcomes by facilitating communication and providing better understanding [77–83].

Length of residency in the host country is one of the most important contributing factors of dental care utilization [36, 38, 42, 48, 51, 54]. Likewise, immigrants who had stayed longer in USA and Canada demonstrated better access to health care and increased service utilization [75]. The association between length of stay and OH was not only limited to OH outcomes, but also positively correlated with OH-related quality of life [35]. The longer immigrants had stayed in the host country, the more likely they had become aware of the health care system and ways to overcome structural barriers to health care such as language, social, or cultural differences [4, 49].

Immigrants’ country of origin was another indicator of acculturation used by a number of studies in our systematic review [4, 53]. Country of origin has been correlated with other aspects of health as well. For instance, a higher prevalence of hypertension was reported among immigrants from Puerto Rico and Dominican Republic compared to Mexican-Americans [84]. Country of origin may reflect immigrants’ cultural background and their attachment to specific beliefs, attitudes, and practices. For example, some cultures have specific diets consisting of high fibre and low refined carbohydrates, or they have defined oral hygiene practices or well-established use of preventive measures such as fluoride [4]. On the other hand, some immigrants are more susceptible to OH problems due to inadequate access to dental care and insurance coverage in their country of origin [4]. In addition, some studies reported that country of origin is one of the most important acculturation measures as it acts as a baseline of immigrants ‘cultural, historical and geographical characteristics that will consequently affect their acculturation level [70, 85, 86]. Biological differences such as genetics, tooth morphology, and oral microflora may also affect the vulnerability of immigrants to OH problems [4]. Moreover, children who were born outside of the United States showed a lower rate of dental visits and higher rate of dental caries compared to their U.S.-born counterparts [50, 52, 55]. Perhaps immigrants’ children born in the host country had better coverage and access to dental care services including school-based programs than their foreign-born counterparts.

In the reviewed studies, age at migration was identified as another acculturation proxy measure affecting immigrants and ethnic minorities’ OH. Those of a younger age at immigration had more advantages than their older counterparts [4, 33, 41, 50, 53, 55]. Migration during old age may be associated with late adaptation to the host country’s services, including the health care system, or perhaps immigrants may not prioritize OH problems over other resettlement issues [4, 53]. Also, preventive dental programs in the host country are usually offered through school programs that are more likely to benefit younger immigrants [41].

Some aspects of OH known to be culturally relevant, like orofacial pain, have been under-reported in the acculturation literature [43] while the association between general pain and acculturation has been widely documented [87–96]. For example, chronic pain was more prevalent among low acculturated South Asians in UK [87]. High pain intensity was reported by low acculturated Chinese Americans in one study [91]; however, in other studies [92, 93] high pain intensity was reported by high acculturated Chinese and Latino American immigrants. Three studies did not find any associations between chronic pain and level of acculturation [88, 94–96]. These inconsistencies may be caused by different study designs, sample characteristics, and interpersonal, cultural, and psychological differences among participants [92]. Moreover, the perception of pain may differ from one culture to another, which could in turn affect the degree of reported pain by immigrants [93]. Acculturation proxies such as language proficiency may also limit immigrants’ ability to understand and respond properly to the questions asked by a health professional. Consequently, this language barrier may lead to inconsistent answers [93].

The quality of the reviewed studies ranged between medium and high. For example, a study conducted among Portuguese-speaking immigrants’ children to assess their caries experience and dental care utilization was attained medium quality due to some reasons such as, unjustified sample size, no description of the response rate or the characteristics of the responders and the non-responders, and no description of the measurement tool [53]. On the other hand, another study investigated the impact of acculturation on Haitian immigrants’ oral health was considered as a high quality as the sample size was justified and satisfactory, a validated measurement acculturation tool was utilized, the study controls for different factors, the statistical test was clearly described and appropriate, and the measurement of the association is presented, including confidence intervals and the probability level (p value) [6].

This study has some limitations that need to be acknowledged. In this review, we included studies that involved immigrant and ethnic groups since it was not possible to distinguish between ethnicity and immigration history (being a newcomer), and we had to rely on the definitions and main categories applied in the included studies [97]. In addition, the diversity among the immigrants at the individual and social level, such as their reasons for immigration, origin and host countries, timing of migration within a political, social environment, and individual life stage makes it hard to lump them together for analysis in one systematic review. Moreover, the restriction to English language among the included studies limited our findings mainly to North America which may penalize information on migratory movements in South America, Africa, Middle East, and Asia. The inconsistency in determining acculturation level and given the quality and quantity of the bibliographic sources identified as a result of the review, does not favor the recommendation of a meta-analysis. While acculturation is an ongoing process of adaptation, most studies included in this review used a cross-sectional design that is unable to show the causal nature of the observed relationships over time. Although the Newcastle-Ottawa Scale was used for quality appraisal of the included studies, there was no validated methodological assessment tool designed specifically for observational studies.

## Conclusion

According to existing evidence, a positive effect of acculturation on OH status and behaviors was found. High acculturated immigrants and etnhic minorities with a longer time of residency in the host country, local language proficiency, and younger age at migration had better OH status and behaviors than their counterparts. Therefore, dental practitioners should be sensitive to cultural differences when providing services to immigrant and ethnic minority groups. Policymakers should also be mindful of cultural barriers and adequately address the unique needs of these individuals to maintain OH equity. Further qualitative and longitudinal studies are needed to better understand acculturation influence on OH. Using validated multidimensional scales instead of acculturation proxies will generate more comprehensive and comparable data. Finally, greater attention should be given to understudied aspects of OH and its association with acculturation.

## Supporting information

S1 Table Search strategy and results from different electronic databases. (PDF)

S2 Table PRISMA 2009 checklist. (PDF)
