# Supplementary material for: Impact of acculturation on oral health among immigrants and ethnic minorities: A systematic review
Source: PLoS One. 2019 Feb 28;14(2):e0212891. doi: 10.1371/journal.pone.0212891 (PMC6395030; doi:10.1371/journal.pone.0212891)
Supplement: S1 Table — (PDF) [file pone.0212891.s001.pdf]

S1 Table. Search strategy and results from different electronic databases

Database	Keywords	Results
PubMed 1976 - Jan 2018	((dental health) OR dentist* OR (periodontal diseases[MeSH Terms]) OR "periodontal diseases" OR (TOOTH DISEASES[MeSH Terms]) OR "tooth disease*" OR (ORAL HEALTH[MeSH Terms]) OR ("oral health")) AND ("cultural adaptation" OR "cultural adequacy" OR "cultural adjustment" OR "cultural affiliation" OR "cultural affiliations" OR "cultural affinities" OR "cultural affinity" OR "cultural affirmation" OR "cultural alienation" OR "cultural assimilation" OR cultural acclimation OR "cultural integration" OR "ethnic identification" OR "ethnic identifications" OR "ethnic identities" OR "ethnic identity" OR social affiliation OR "social affiliation" OR "social affiliations" OR "social affinities" OR "social affinity" OR "country of birth" OR "country of origin" OR "language preference" OR "language preferences" OR "language preferred" OR "language preferring" OR language competence OR "language competence" OR "language competences" OR "language competencies" OR "language competency" OR "language proficiency" OR age of arrival OR acculturation[MeSH Terms] OR "acculturation*" OR "adaptation" OR "assimilation*" OR "enculturation*" OR marginaliz* OR marginalis* OR "transculturated" OR "transculturation" OR "transculture" OR "transcultural" OR social identification[MeSH Terms] OR "social identification" OR "social identifications" OR "social identities" OR "social identity" OR "nativity") AND (immigrants[MeSH Terms] OR "immigrant*" OR Emigrants[MeSH Terms] OR "emigrant*" OR "ethnic groups" OR Emigration[MeSH Terms] OR immigration[MeSH Terms] OR "emigration" OR "immigration" OR "transients" OR migrant* OR immigrat* OR immigrant)	249
Ovid MEDLINE 1983 - Jan 2018	"(adapt* adj2 cultur*).mp. OR (adequa* adj2 cultur*).mp.OR (cultur* adj2 adjust*).mp. OR exp Acculturation/ OR Acculturat*.mp. OR (cultur* adj2 affiliat*).mp. OR (cultur* adj2 affinit*).mp. OR (cultur* adj2 affirm*).mp. OR (cultur* adj2 alienat*).mp. OR transculturat*.mp. OR (cultur* adj2 assimilat*).mp. OR (cultur* adj2 acclimat*).mp. OR (cultur* adj2 integrat*).mp. OR exp Social Identification/ OR (ethnic* adj2 identif*).mp. OR (social adj2 affiliat*).mp. OR nativity.mp. OR (countr* adj1 birth).mp. OR (language* adj1 prefer*).mp. OR (language adj1 competenc*).mp. OR (language adj2 proficiency).mp. OR age of arrival.mp." AND "exp "Emigrants and Immigrants"/ OR Ethnic Groups/ OR immigrant*.mp. OR exp "Emigration and Immigration"/ OR immigrat*.mp. OR "Transients and Migrants"/ OR migrant*.mp." AND "Oral Health/ OR oral health.mp. OR exp Tooth Diseases/ OR exp Periodontal Diseases/ OR dentist*.mp. OR exp Dental Health Services/"	58
PsychInfo 2008 - Jan 2018	"(adapt* adj2 cultur*).mp. OR (adequa* adj2 cultur*).mp.OR (cultur* adj2 adjust*).mp. OR exp Acculturation/ OR Acculturat*.mp. OR (cultur* adj2 affiliat*).mp. OR (cultur* adj2 affinit*).mp. OR (cultur* adj2 affirm*).mp. OR (cultur* adj2 alienat*).mp. OR transculturat*.mp. OR (cultur* adj2 assimilat*).mp. OR (cultur* adj2 assimilat*).mp. OR (cultur* adj2 acclimat*).mp. OR (cultur* adj2 integrat*).mp. OR social identity/ OR (ethnic* adj2 identif*).mp. OR (social adj2 affiliat*).mp. OR nativity.mp. OR (countr* adj1 birth).mp. OR (language* adj1 prefer*).mp. OR (language adj1 competenc*).mp. OR (language adj2 proficiency).mp. OR age of arrival.mp." AND "Ethnic Groups/ OR immigrant*.mp. OR immigrat*.mp. OR "Transients and Migrants"/ OR migrant*.mp." AND "Oral Health/ OR oral health.mp. OR dentist*.mp. OR tooth disease*.mp. OR periodont* disease*.mp."	8
Sociological Abstracts 1994 - Jan 2018	(((all(oral health)) OR (all(tooth disease*)) OR (all(periodontal disease*)) OR (all(dental health)) OR all(dentist*)) OR SU.EXACT("Dental Care")) AND (((all(Ethnic Group*)) OR (all(immigrant*)) OR (all(immigrat*)) OR (all(migrant*)) OR (all(emmigrant*)) OR (all(emigrat*))) OR (SU.EXACT("Immigration") OR SU.EXACT("Emigration"))) AND (((all(adapt*) NEAR/2 all(cultur*)) OR (all(adequa*) NEAR/2 all(cultur*)) OR (all(cultur*) NEAR/2 all(adjust*)) OR (all(Acculturat*)) OR (all(cultur*) NEAR/2 all(affiliat*)) OR (all(cultur*) NEAR/2 all(affinit*)) OR (all(cultur*) NEAR/2 all(affirm*)) OR (all(cultur*) NEAR/2 all(alienat*)) OR (all(transculturat*)) OR (all(cultur*) NEAR/2 all(assimilat*))) OR ((all(cultur*) NEAR/2 all(acclimat*)) OR (all(cultur*) NEAR/2 all(integrat*)) OR (all(ethnic*) NEAR/2 all(identif*)) OR (all(social) NEAR/2 all(affiliat*)) OR (all(nativity)) OR (all(countr*) NEAR/1 all(birth)) OR (all(language*) NEAR/1 all(prefer*)) OR (all(language) NEAR/1	23

	all(competenc*)) OR (all(language) NEAR/2 all(proficien*)) OR (all(age of arrival))) OR (SU.EXACT("Acculturation") OR SU.EXACT("Social Identity"))	
Embase 1979 - Jan 2018	"(adapt* adj2 cultur*).mp. OR (adequa* adj2 cultur*).mp. OR (cultur* adj2 adjust*).mp. OR Acculturat*.mp. OR (cultur* adj2 affiliat*).mp. OR (cultur* adj2 affinit*).mp. OR (cultur* adj2 affirm*).mp. OR (cultur* adj2 alienat*).mp. OR transculturat*.mp. OR (cultur* adj2 assimilat*).mp. OR (cultur* adj2 acclimat*).mp. OR (cultur* adj2 integrat*).mp. OR Social Identification.mp. OR (ethnic* adj2 identif*).mp. OR (social adj2 affiliat*).mp. OR nativity.mp. OR (countr* adj1 birth).mp. OR (language* adj1 prefer*).mp. OR (language adj1 competenc*).mp. OR (language adj2 proficiency).mp. OR age of arrival.mp" AND " Emigrant*.mp. OR immigrant*.mp. OR immigrat*.mp. OR migrant*.mp. OR exp migrant/ OR transient.mp. OR exp ethnic group/" AND " oral health.mp. OR exp Tooth Diseases/ OR exp Periodontal Diseases/ OR dentist*.mp. OR dental health services.mp."	116
CINAHL 1989 - Jan 2018	" (MH"Tooth diseases+") OR (MH"oral health") OR oral health OR periodontal disease* OR (MH"Periodontaldiseases+") OR Dentist* OR (MH"Dentists") OR (MH"Dental health services+") OR Dental health service* OR tooth disease*" AND "(MH"emigration and immigration") OR (MH"Immigrants") OR immigrant* OR ethnic group* OR immigrat* OR (MH"transients and Migrants") OR transient* OR migrant* OR emigrant* OR emigration and immigration " AND " adapt* N2 cultur* OR adequa* N2 cultur* OR cultur* N2 adjust OR Acculturat* OR cultur* N2 affiliat* OR cultur* N2 affinit* OR cultur* N2 affirm* OR cultur* N2 alienat* OR transculturat OR cultur* N2 assimilat* OR cultur* N2 acclimat* OR cultur* N2 integrat* OR social identification OR ethnic* N2 identif* OR social N2 affiliat* OR nativity OR countr* N1 birth OR language* N1 prefer* OR language N1 competenc* OR language N2 proficiency OR age of arrival "	76
Web of Science 1995 - Jan 2018	"TOPIC: ((adapt* NEAR/2 cultur*)) OR TOPIC: ((adequa* NEAR/2 cultur*)) OR TOPIC: ((cultur* NEAR/2 adjust*)) OR TOPIC: (Acculturat*) OR TOPIC: (cultur* NEAR/2 affiliat*) OR TOPIC: ((cultur* NEAR/2 affinit*)) OR TOPIC: (cultur* NEAR/2 affirm*) OR TOPIC: ((cultur* NEAR/2 alienat*)) OR TOPIC: (transculturat*) OR TOPIC: ((cultur* NEAR/2 assimilat*)) OR TOPIC: ((cultur* NEAR/2 acclimat*)) ORTOPIC: ((cultur* NEAR/2 integrat*)) OR TOPIC: ((ethnic* NEAR/2 identif*)) OR TOPIC: ((social NEAR/2 affiliat*)) OR TOPIC: (nativity) OR TOPIC: ((countr* NEAR/1 birth).) OR TOPIC: ((language* NEAR/1 prefer*)) OR TOPIC: ((language NEAR/1 competenc*)) OR TOPIC: ((language NEAR/2 proficiency)) OR TOPIC: (age of arrival) " AND "TS=(Ethnic Groups) OR TS=(immigrant*) OR TS=(immigrat*) OR TS=(Transients and Migrants) OR TS=(migrant*) OR TS=(Emigrants and Immigrants) OR TS=(Emigration and Immigration)" AND "TOPIC: (Oral Health) OR TOPIC: (oralhealth.mp.) OR TOPIC: (ToothDiseases) OR TOP IC: (PeriodontalDiseases) OR TOPIC: (dentist*) OR TOPIC: (Dental Health Services)	111
Total databases searches		641
Duplicates		240
Final		401
